# Evaluation of video background and stimulus transparency in a visual ERP-based BCI under RSVP

**DOI:** 10.1007/s11517-025-03498-5

**Published:** 2026-01-10

**Authors:** Álvaro Fernández-Rodríguez, Francisco Velasco-Álvarez, Francisco-Javier Vizcaíno-Martín, Ricardo Ron-Angevin

**Affiliations:** 1 Departamento de Psicología Básica, Universidad de Málaga, 29071 Malaga, Spain; 2 Departamento de Tecnología Electrónica, Universidad de Málaga, 29071 Malaga, Spain

**Keywords:** Brain-computer interface (BCI), Event-related potential (ERP), Rapid serial visual presentation (RSVP), Stimulus, Background, Transparency

## Abstract

Rapid serial visual presentation (RSVP) is a promising paradigm for visual brain–computer interfaces (BCIs) based on event-related potentials (ERPs) for patients with limited muscle and eye movement. This study explores the impact of video background and stimulus transparency on BCI control, factors that have not been previously examined together under RSVP. Two experimental sessions were conducted with 12 participants each. Four BCI conditions were tested: opaque pictograms, and white background (A255W); opaque pictograms, and video background (A255V); intermediate transparent pictograms, and video background (A085); and highly transparent pictograms, and video background (A028V). The results indicated that the video background had a negative impact on BCI performance. In addition, the intermediate transparent pictograms (A085V) proved to be balanced, as it did not show significant performance differences compared to opaque pictograms (A255V) but was rated significantly better by users on subjective measures related to attending to the video background. Therefore, in applications where users must shift attention between BCI control and their surroundings, balancing stimulus transparency is a suitable option for enhancing system usability. These findings are particularly relevant for designing asynchronous ERP-BCIs using RSVP for patients with impaired oculomotor control.

**Graphical abstract:**

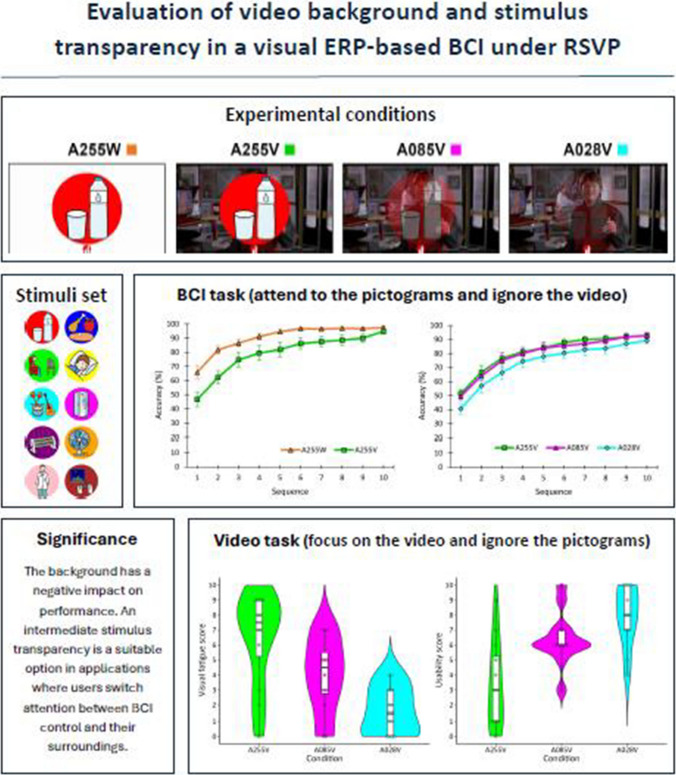

**Supplementary Information:**

The online version contains supplementary material available at 10.1007/s11517-025-03498-5.

## Introduction

Brain–computer interfaces (BCIs) are systems that serve as assistive technology (AT), allowing users to interact with their environment directly through their brain signals [1]. The primary goal of AT is to enhance user interaction with their surroundings [2]. Alongside BCIs, various other tools can be utilized as AT, including eye trackers, head-pointing devices, and low-pressure sensors. However, specific conditions such as amyotrophic lateral sclerosis (ALS) can severely impair both muscular and ocular control [3], rendering certain types of AT ineffective. In cases of severe motor limitations, BCIs offer a promising alternative, as they do not initially rely on the muscular channel for control.

While BCI systems can utilize various types of brain signals, electroencephalographic (EEG) activity—particularly sensorimotor rhythms (SMRs), steady-state visual evoked potentials (SSVEPs), code-modulated visual evoked potentials (c-VEPs), and event-related potentials (ERPs)—is widely employed due to its portability, cost-effectiveness, and high temporal resolution (see [4], for a review of EEG-based BCIs). ERPs, in particular, are extensively used for controlling specific applications without requiring ocular mobility [5] and are therefore considered highly suitable for BCI control in cases of severe motor impairment. ERPs reflect changes in brain electrical activity elicited by specific events, often presented through visual stimuli (see [6], for a review of ERP-BCIs). The visual modality is generally preferred in ERP-BCIs because of its reliability and adaptability, even among users with limited or no eye movement. For instance, rapid serial visual presentation (RSVP) paradigms have proven especially valuable for enabling ERP-BCI control without depending on ocular mobility (e.g., [7, 8]).

In RSVP, visual stimuli are presented sequentially in the same spatial location, requiring the user to focus on a specific stimulus, such as a pictogram related to their intention. The fact that the spatial location of all stimuli is the same means that the system does not depend on the user’s ability to move their eyes; however, it is necessary for the person to be able to focus and visually differentiate what is being observed. The aim of an ERP-BCI is to differentiate between the brain signals generated in response to attended (target) and unattended (non-target) stimuli, with particular emphasis on the P300 component. The P300 is a positive deflection that occurs 300–600 ms after the presentation of the expected stimulus [9]. However, ERP-BCI applications often consider a wider range of ERPs beyond this period, including components like P200, N200, and the late positive potential. Any signal that helps distinguish the attended stimulus (target) from the unattended stimuli (non-targets) is incorporated within the relevant time window, typically spanning from 0 to 800 ms after stimulus onset (e.g., [10, 11]).

The target population for these interfaces often has such limited motor capabilities that they are unable to communicate to the caregiver when they want to use the interface. As a result, these individuals may require an interface that delivers stimuli continuously within their field of vision, enabling the system to asynchronously detect when they wish to select a command (e.g., [12]). This continuous presentation approach addresses the challenge of how a caregiver would know when the user wants to use the interface. Previous studies have utilized BCIs for applications that ideally toggle between control and non-control periods, such as in home automation systems or wheelchairs (e.g., [13, 14]). To adapt these applications to an ERP-BCI using RSVP, visual stimuli must be constantly presented within the user’s field of vision. However, this continuous presentation of stimuli can create challenges for both interface control and the user’s ability to attend to their surroundings.

In visual attention research, it is well established that irrelevant visual input can impair task performance. According to load theory [15], attentional resources are limited, and distractors such as moving backgrounds may compete with target processing, thereby reducing detection efficiency. Empirical evidence supports this view: visual distractors embedded within RSVP streams have been shown to exacerbate the attentional blink and reduce target identification [16], while dynamic video backgrounds, compared to static monochromatic ones, further decrease detection rates and P300 amplitudes [17]. These findings are especially relevant for ERP-based BCIs, as the robustness of the P300 component is highly sensitive to attentional load. This raises the question of whether dynamic backgrounds could negatively affect the performance of visual ERP-BCIs under RSVP in detecting user-intended stimuli. Some studies have explored ERP-BCIs in dynamic visual contexts, such as wheelchair navigation, video game control or home automation (e.g., [14, 18, 19], respectively). However, to our knowledge, only Kim et al. [20] have directly examined the effect of background video on visual ERP-BCI performance, reporting a negative impact under video conditions. Importantly, their system employed only four commands located in different screen positions, requiring oculomotor control and not relying on RSVP. This gap underscores the need to investigate how dynamic backgrounds influence ERP-BCIs specifically under RSVP paradigms.

Furthermore, a visual ERP-BCI overlaid on a dynamic background could negatively impact the user experience when the user wishes to attend to the background. One potential solution is to use semi-transparent stimuli that allow both the effective use of the visual ERP-BCI and perception of the background. However, increasing the transparency of the stimuli could also amplify the negative impact on ERP-BCI performance, as it makes the video background more perceptible and potentially more distracting. According to studies by Li et al. [21] using a gaze-dependent row-column paradigm, and Lian et al. [22] using RSVP, luminosity contrast—which decreases with increasing transparency—has been shown to positively influence performance. Therefore, it can be suggested that increased transparency of the stimuli may impair ERP-BCI performance by reducing the contrast against a dynamic background, compared to more opaque stimuli.

In summary, this study investigates how dynamic video backgrounds and varying levels of stimulus transparency affect both performance and user experience in controlling a visual ERP-BCI under RSVP. Beyond addressing these experimental questions, the findings are intended to guide the development of ERP-BCIs that are not only clinically useful but also ecologically valid. This is particularly relevant for multimedia applications (e.g., television viewing or web browsing) and emerging augmented reality (AR) contexts, where digital stimuli must coexist with continuously changing visual environments. Striking the right balance between transparency and system robustness is therefore essential to ensure that ERP-BCIs remain practical, unobtrusive, and seamlessly integrated into everyday life. Ultimately, these advances are expected to benefit patients with severe motor impairments, for whom continuously available and user-friendly BCI systems could provide a vital means of interaction and communication.

## Materials and methods

### Participants

The complete study consisted of two experimental sessions involving different participants. Session 1 involved 12 able-bodied participants (aged 22 ± 5.49 years, 5 females, 7 males). Session 2 also involved 12 able-bodied participants (aged 25.91 ± 13.55 years, 2 females, 10 males). All participants had normal or corrected-to-normal vision and were of legal age. The study was approved by the Ethics Committee of the University of Malaga and adhered to the ethical standards set forth in the Helsinki Declaration. According to self-reports, none of the participants had a history of neurological or psychiatric illness. All participants provided written informed consent.

### Data acquisition and signal processing

EEG data were recorded at a sample rate of 250 Hz using electrode positions Fz, Cz, Pz, Oz, P3, P4, PO7, and PO8, according to the 10/10 international system. This subset of electrodes covers parietal–occipital and central regions, which have been widely linked to the detection of ERP components in BCI applications [23]. Furthermore, limiting the number of electrodes reduces computational load and facilitates future applications in practical BCI systems, where ease of setup and user comfort are essential. All channels were referenced to the right mastoid and grounded to position AFz. Impedance was reduced to below 10 kΩ for each electrode with electrolyte gel. Signals were amplified by an actiCHamp amplifier (Brain Products GmbH, Gilching, Germany). Data acquisition was conducted using the BCI2000 software (v3.6) [24]. To optimize signal quality, recordings were band-pass filtered between 0.1 and 9 Hz (a first-order infinite impulse response high-pass filter and a second-order Butterworth infinite impulse response low-pass filter), which attenuated slow drifts and high-frequency muscle activity. In addition, a notch filter at 50 Hz (two third-order Chebyshev filters) was applied to suppress interference from power lines. After data acquisition, remaining artifacts were corrected using the artifact subspace reconstruction (ASR) algorithm in EEGLAB (v2024.0.0) with default parameters and the Riemannian distance [25, 26]. This preprocessing pipeline, which combined filtering and ASR, provided robust attenuation of powerline, muscular, and cardiac artifacts while preserving the relevant ERPs. Subsequently, a stepwise linear discriminant analysis of the data was performed to obtain the weights of the classifier and to calculate the accuracy (using the BCI2000 tool called *P300Classifier*).

### Experimental conditions

**Fig. 1:**
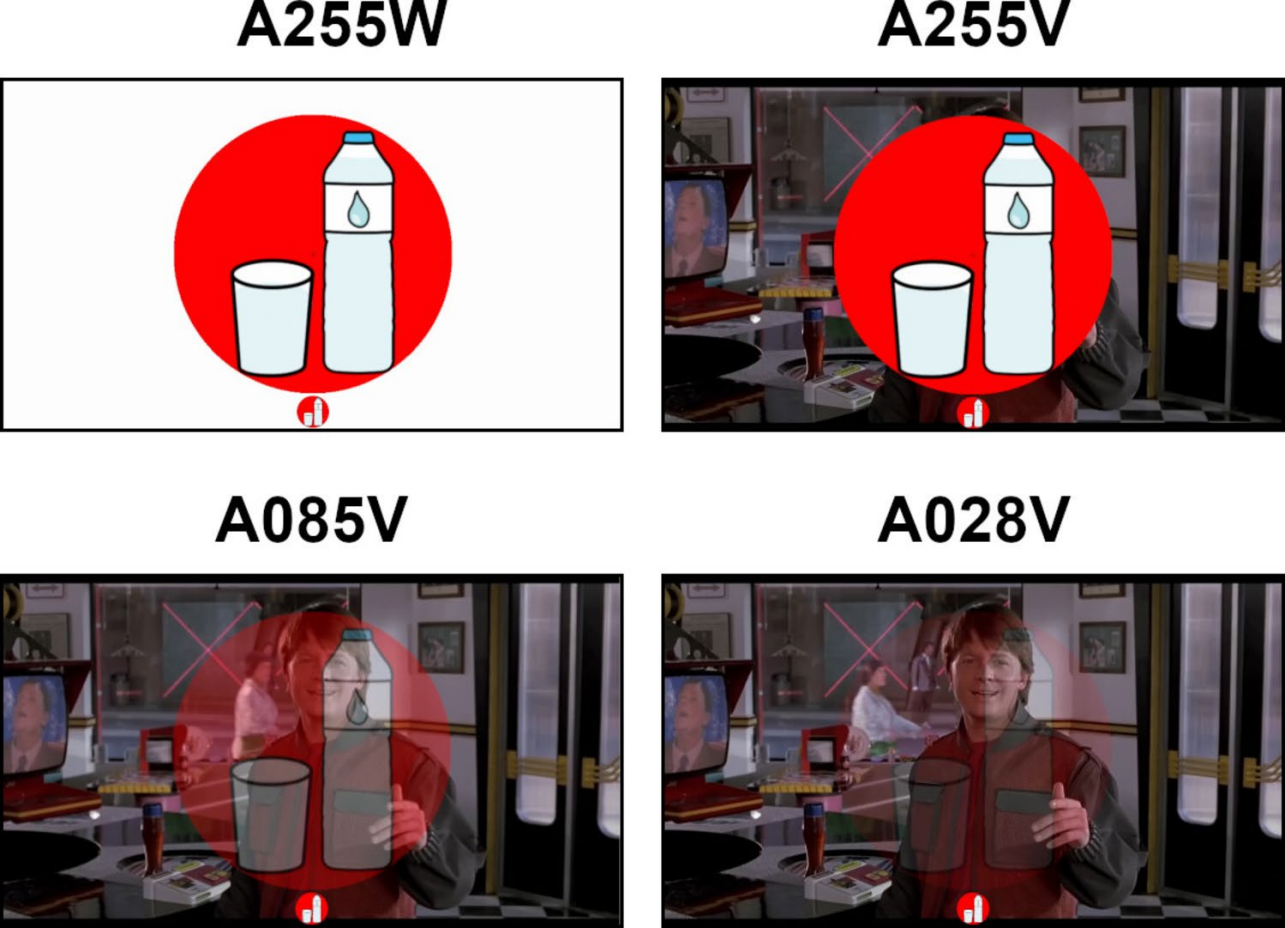
Screenshot displaying the appearance of one of the pictograms (water with the red circle) under different conditions (A255W, A255V, A085V, and A028V, where the number denotes the alpha channel level of the pictogram) with scene 1 (*Back to the Future. Part II*, the hoverboard scene) as the background

The application used in the present study was a visual ERP-BCI, with control commands represented by pictograms that could be selected through an attention task. Specifically, the task required participants to count the number of times the desired pictogram appeared on the screen while ignoring the others. Various conditions were tested for this study (Fig. 1). The experimental conditions were as follows: (i) opaque pictograms with alpha channel set to 255 units, and white background (A255W); (ii) opaque pictograms with alpha channel set to 255 units, and video background (A255V); (iii) semi-transparent pictograms with alpha channel set to 85 units, and video background (A085V); (iv) and semi-transparent pictograms with alpha channel set to 28 units, and video background (A028V).

**Fig. 2:**
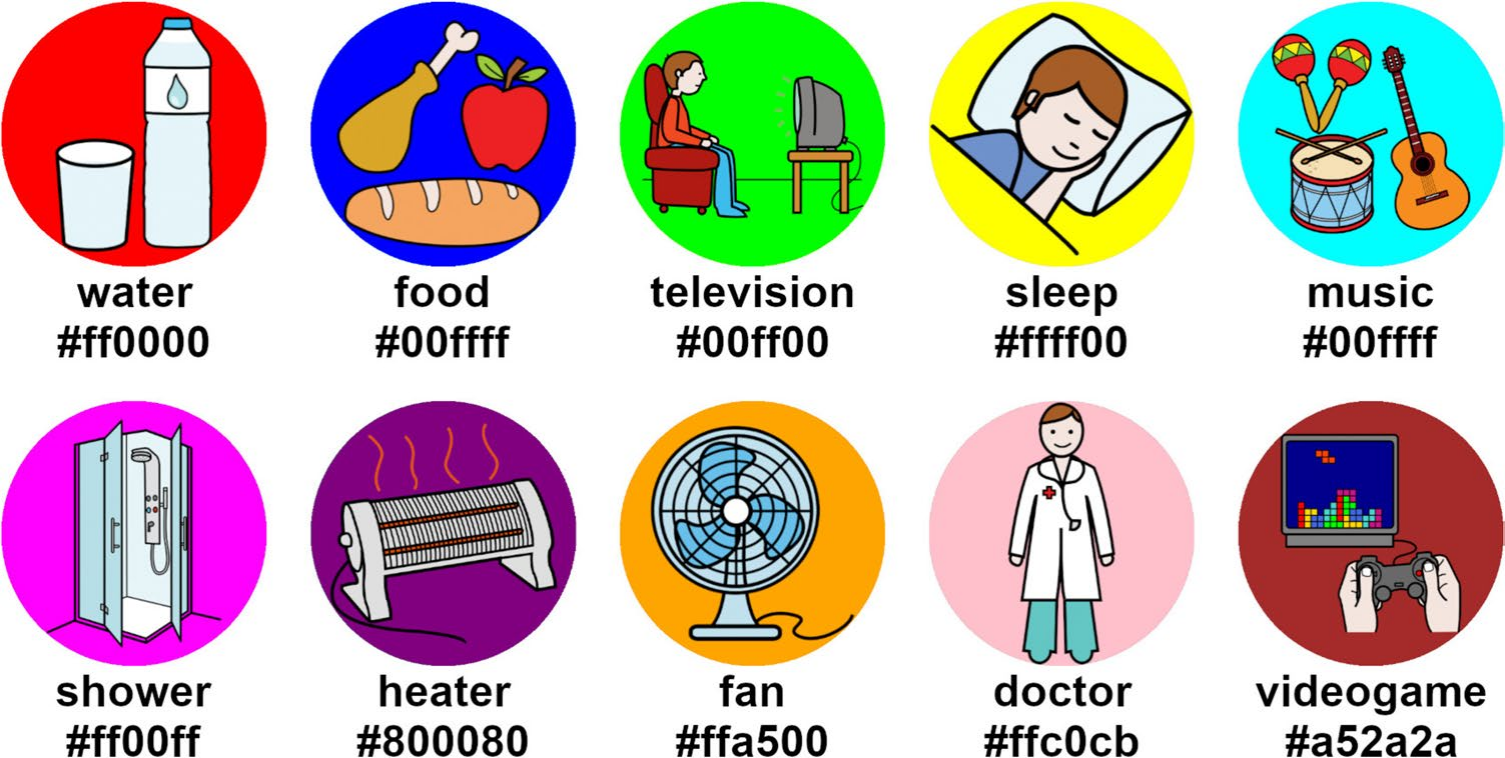
Each pictogram in the experiment labeled with its meaning and circle’s color in HTML format

The pictograms used for these conditions were obtained from the database of the Centro Aragonés para la Comunicación Aumentativa y Alternativa (ARASAAC) (https://arasaac.org). Pictograms were chosen as they could serve as suitable stimuli to represent possible messages or actions that a user might want to select, similar to those used in alternative and augmentative communication (AAC) applications [27]. Each condition used the same set of 10 distinct pictograms, each with a uniquely colored circle behind it to facilitate visual discrimination (Fig. 2). These stimuli were presented under the RSVP paradigm, meaning they all appeared in the same position—in the center of the screen—with a diameter of ~ 15.53 cm (864 px), for 180 ms, with an interstimulus interval (ISI) of 100 ms, resulting in a stimulus onset asynchrony (SOA) of 280 ms. The background videos used for A255V, A085V, and A028V consisted of three different movie clips: scene 1 from Back to the Future Part II (a bright and dynamic scene where Marty McFly escapes on a hoverboard, starting at approximately 00:17:15) [28]; scene 2 from Split (a dark, tense scene in which the character Casey wakes up after being kidnapped, starting at approximately 01:00:23) [29]; and scene 3 from Green Book (a bright, calm scene depicting a hot-dog eating contest in a bar, starting at approximately 00:09:55) [30]. While the starting point of each video was always the same across tasks, the endpoint varied depending on the duration of each task (see Sect. 2.5 Procedure). These scenes were chosen to provide varied visual dynamics and lighting conditions while maintaining high image quality and aiming to promote participant engagement. Due to copyright restrictions, we cannot share the original videos used in the study.

Due to the limitations of BCI2000 in modifying the display as required for this experiment, the Processing (v4.3) software was used to present the stimuli for each condition with the background video [31]. Processing is a graphics software coded in Java that was synchronized in time with BCI2000 through a UDP port (using the BCI2000 watches tool) and received the temporal instant in which the target stimulus was presented in BCI2000.

A Lenovo Legion 5 15IAH7H laptop was used (15.6", Intel Core i7-12700H, 16 GB RAM, GeForce RTX 3060, Windows 11 Home) at a resolution of 1920 × 1080 px. The refresh rate of the screen was 144 Hz. The distance between the user’s point of view and the screen was ~ 50 cm.

### Experimental tasks

**Fig. 3:**
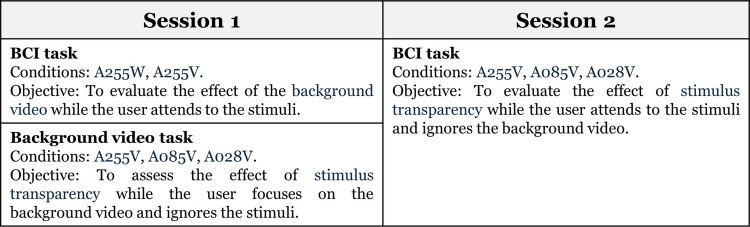
Summary of the tasks performed in each experimental session: in Session 1, the brain-computer interface (BCI) task with A255W and A255V, and the background video task with A255V, A085V and A028V; in Session 2, only the BCI task with A255V, A085V, and A028V

The experiment comprised two independent sessions: Session 1 and Session 2 (Fig. 3). In Session 1, two types of tasks were performed: a BCI task, with conditions A255W and A255V, aimed at evaluating the effect of the background video when the user attended to the pictogram; and a background video task, with conditions A255V, A085V, and A028V, intended to assess the effect of pictogram transparency when the user focused on the video and ignored the pictograms. In Session 2, only the BCI task was performed, with conditions A255V, A085V, and A028V, to evaluate the transparency effect of the stimuli while the user attended to the pictograms and ignored the background video. Details of these tasks are provided below.

#### Brain–computer interface task

The aim of the BCI task was to evaluate the system’s performance in discriminating between the ERP signal associated with the target stimulus and the non-target ones in each of the experimental conditions. This task was conducted with conditions A255W and A255V in Session 1, and with conditions A255V, A085V, and A028V in Session 2. Regardless of the condition, the requirements for the participant were the same. The user had to focus their attention on the indicated pictogram (i.e., the target stimulus) while keeping their gaze fixed on a small cross in the center of the screen to simulate the absence of oculomotor control. The user did not receive online feedback since it was an exclusively offline BCI task, and their performance was calculated once the session had ended.

**Fig. 4:**
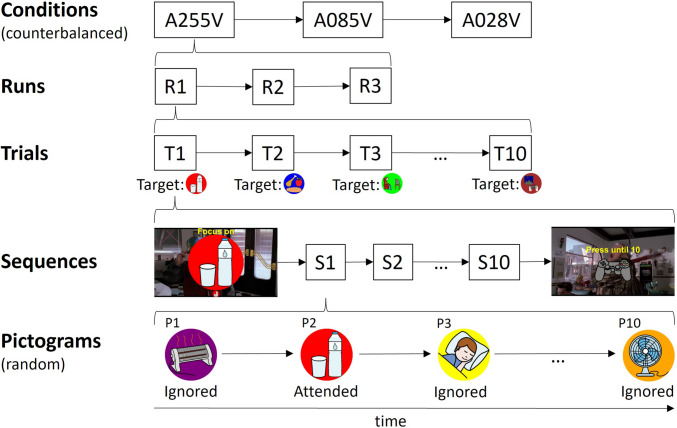
Experimental procedure followed by the participants in the brain-computer interface (BCI) tasks for Session 2. The procedure for Session 1 was similar, except with two conditions (A255W and A255V) instead of three. The order of the conditions was counterbalanced between different participants. Likewise, the order of presentation of the stimuli in each sequence was random, without replacement

The process of selecting a pictogram in the interface, from the 10 possible options, was referred to as a trial (Fig. 4). During a trial, each pictogram was presented 10 times. The presentation of each pictogram available on the interface corresponded to a sequence, and thus, a trial was composed of 10 sequences. The order of presentation of the pictograms in each sequence was random, without replacement. The user was asked to mentally count the number of presentations of the target pictogram to ensure their attention was focused on the task. Ideally, during a trial, the user was supposed to count the presentation of 10 target stimuli; however, it was possible for some to be missed. To record the number of stimuli that had been missed, the user had to press a button on a game controller at the end of the trial as many times as the number of stimuli they missed.

Before starting each trial, there was a pause of 5 s. At the beginning of this pause, the pictogram to be attended to in the next trial was indicated by the message “Focus on”, and the target pictogram was shown simultaneously in the same position for 4 s. At the end of the trial—i.e., after all sequences had finished—there was another pause of 4 s, during which an image of a game controller with the text “Press until 10” was presented to remind the participant to press the button on the game controller and count up to 10, with the goal of easily registering the number of target pictograms they had missed. The duration of a single trial was 27.9 s: 100 stimulus presentations (10 sequences × 10 pictograms) of 180 ms each, with 99 ISIs of 100 ms. The background video was only played during the trial, meaning it was paused during the “Focus on” and “Press until 10” periods.

A run was defined as the set of trials from when the application was started by the experimenter until it was automatically stopped. Each run consisted of 10 selections (trials), with 10 different pictograms focused on. The order of pictogram selection was always the same, as depicted in Fig. 2 in row-major order. For each condition, 3 runs were carried out one after another (30 trials per condition, 3 runs × 10 trials). The order of the conditions was also counterbalanced—i.e., evenly distributed—across participants to prevent any unwanted effects such as learning or fatigue, ensuring all conditions were equally represented.

#### Background video task

The purpose of the background video task was to evaluate the perceived level of annoyance experienced by users when watching a movie scene while pictograms were presented under RSVP. In this task, which no longer required the use of EEG, participants watched a movie scene with pictograms displayed at three levels of transparency: A255V, A085V, and A028V. The same videos used in the BCI tasks were employed. However, unlike in the BCI task, the videos were not paused, and the pictograms appeared continuously, with no interruptions such as “Focus on” or “Press until 10” pauses. Participants were instructed to ignore the pictograms and focus on the background video. Each transparency condition was tested once, with a single run per condition. The order of the transparency conditions was counterbalanced across participants, while the sequence of the background videos remained consistent. For instance, for participant BU01, the conditions and scenes were A255V-scene 1, A085V-scene 2, and A028V-scene 3, whereas for BU02, the order was A255V-scene 1, A028V-scene 2, and A085V-scene 3. In each run, a total of 970 presentations were made (10 different pictograms × 97 times each), with the same timing as in the BCI task (pictogram displayed for 180 ms, ISI of 100 ms). Thus, each run lasted ~ 272 s. After completing the three runs, participants filled out a questionnaire to assess their subjective experience during the video viewing under each condition.

### Procedure

**Fig. 5:**
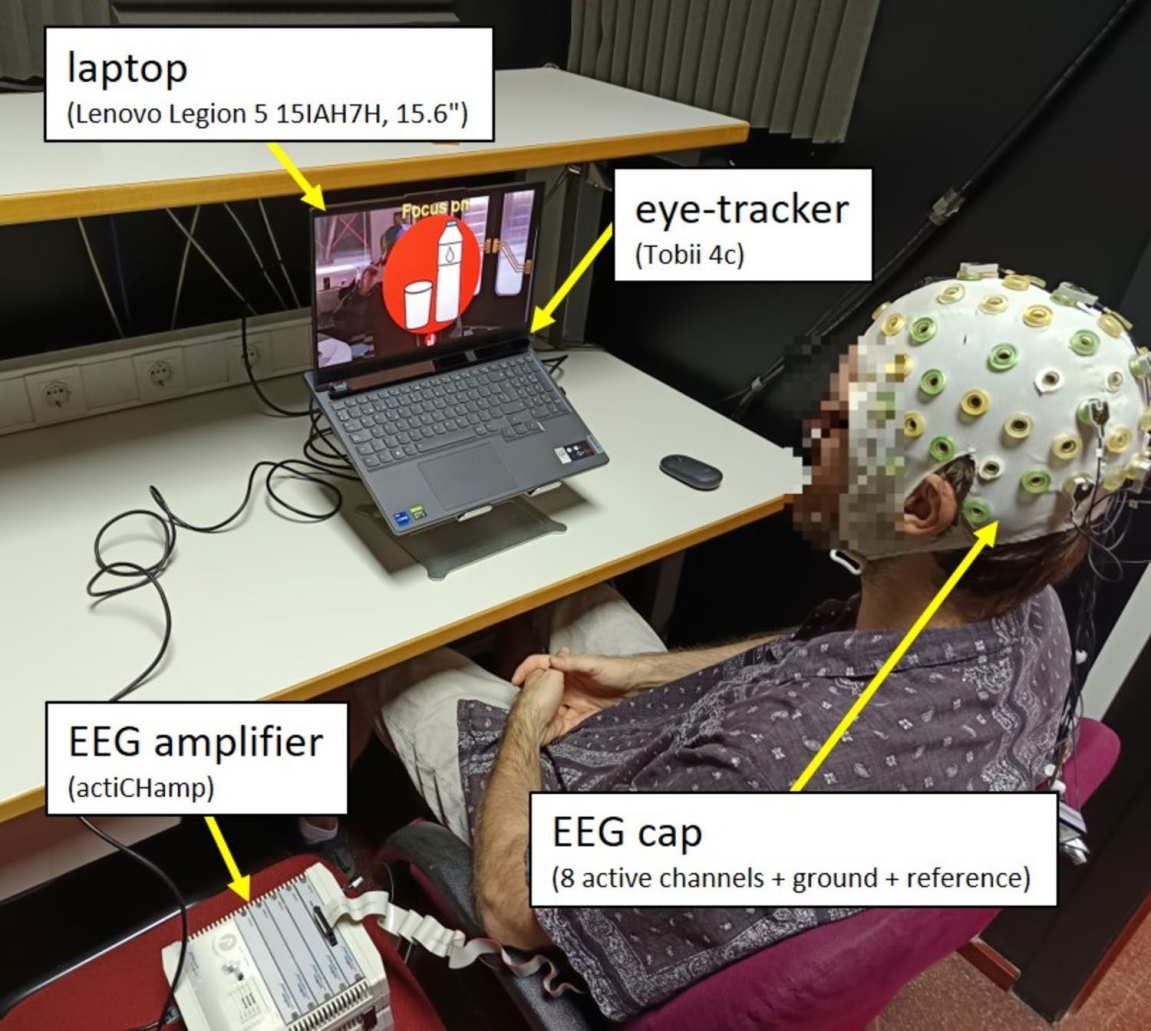
Overview of the experimental setup during the RSVP-BCI task, including the participant, display, and recording equipment

Due to the number of conditions and experimental tasks involved, the study was divided into two sessions (Session 1 and Session 2), with different participants for each session. Within each session, an intra-subject design (also known as a repeated measures design) was used, meaning that all participants were exposed to all conditions. The initial procedure for each session was consistent: upon arrival at the laboratory, the test was explained to the participants, they signed an informed consent form, and the necessary instrumentation was set up (Fig. 5). After these preparations, the specific tasks differed between the sessions, as detailed below.

#### Session 1

In Session 1, participants completed the BCI task using A255W and A255V, and the background video task using A255V, A085V, and A028V. First, in the BCI task, the variable manipulated was the type of background: a static white background (A255W) versus a movie scene (A255V). The goal of this comparison was to assess the impact of having a background video. After each condition, participants completed a questionnaire to evaluate their experience. The order of the conditions was counterbalanced across participants. Second, in the background video task, the variable manipulated was the transparency of the pictograms. This comparison aimed to explore the potential benefit of using semi-transparent stimuli (A028V and A085V) versus opaque ones (A255V) while participants focused on a background video. As with the BCI task, participants filled out a questionnaire after each condition, and the order of the three conditions was counterbalanced among participants. The total duration of Session 1 was approximately 100 min, from the participant’s arrival at the laboratory until all tasks were completed.

#### Session 2

In Session 2, participants completed only the BCI task under the three conditions with a background video: A255V, A085V, and A028V. This BCI task was specifically designed to evaluate the effects of different levels of pictogram transparency while a background video was present. It complemented the background video task from Session 1, which assessed user preferences when focusing on the video while ignoring the pictograms. By conducting both tasks (BCI and background video), we obtained a comprehensive evaluation of the conditions (A255V, A085V, and A028V) in both scenarios: when users focused on the video and when they needed to select pictograms. After each condition, participants completed a questionnaire to assess various aspects of their subjective experience. The order of conditions was counterbalanced across participants. The total duration of Session 2 was approximately 110 min, from the participant’s arrival at the laboratory until the completion of all tasks.

### Evaluation

To explore the effect of the background video and stimulus transparency, several variables were collected for each of the experimental tasks: (i) BCI performance, (ii) user target detection, (iii) event-related potential waveform, (iv) subjective items, and (v) gaze position on the screen. Each of these variables will be detailed below. Additionally, the statistical analyses used to evaluate these variables will be explained.

#### Brain-computer interface performance

To assess BCI performance in each condition, two parameters were used: (i) accuracy (%), which indicates the percentage of correctly classified selections out of the total predicted selections, and (ii) ITR (bit/min), which objectively measures the system’s information rate [32]. The ITR calculation considers the accuracy (*P*), the number of commands in the interface (*N*), and the time for identification (*T*):

$$ITR=\left[{\mathrm{log}}_{2}N+P{\mathrm{log}}_{2}P+\left(1-P\right){\mathrm{log}}_{2}\left(\frac{1-P}{N-1}\right)\right]\cdot \frac{60}{T}$$

These parameters were computed offline for each trial sequence (from 1 to 10), allowing the examination of performance progression throughout the trial.

#### Target detection

During the BCI tasks, users’ ability to perceive stimuli was assessed by the percentage of correctly identified target stimuli in each trial. The user was informed that each pictogram would be presented 10 times throughout the trial and was instructed to mentally count each appearance of the target stimulus. At the end of the trial, users were to continue their mental count up to 10, pressing a button on a game controller for each target stimulus they had missed. For instance, if a user counted 8 presentations of the target stimulus, they would press the button 2 times. This measure indicated the number of presentations the user missed and, therefore, provided their accuracy in recognizing the target stimuli.

#### Event-related waveform

The amplitude (µV) of the ERP waveform was analyzed during the BCI tasks to assess the impact of stimulus background and transparency on both target and non-target stimuli. This analysis covered a time window from − 200 to 1000 ms, with the interval from − 200 to 0 ms serving as the baseline. The dependent variable used to compare the ERP waveform for each condition was the amplitude difference between target and non-target stimuli. However, the grand averages for target and non-target stimuli are presented in the supplementary material. The amplitude difference helps eliminate the presence of visual evoked potentials (VEPs) and highlights components specifically related to the presentation of the stimulus desired by the user. Additionally, the amplitude difference is likely a useful variable for analyzing classifier performance, as a greater difference between the signals associated with target and non-target stimuli can make it easier for the classifier to accurately distinguish between them [33].

#### Subjective items

The experimental conditions were also evaluated using several Likert-scale items—from 0 (strongly disagree) to 10 (strongly agree)—to assess user experience during each task: the BCI tasks in Session 1 (A255W and A255V) and Session 2 (A255V, A085V, and A028V), and the background video tasks in Session 1 (A255V, A085V, and A028V).

First, to evaluate the background effect in the BCI tasks (Session 1), participants answered the following items after each of the two conditions (A255W and A255V):

1. It has been easy for me to recognize the target pictogram. [*perception*]
2. Paying attention to the pictograms has caused visual fatigue. [*visual fatigue*]
3. I have found the chosen background to be comfortable. [*comfort*]
4. If I were a patient with severe motor limitations, I would be willing to use this condition for extended use of the interface. [*usability*]

Secondly, to evaluate the effect of stimulus transparency in the BCI task (Session 2), participants answered the following items after each of the three conditions (A028V, A085V, and A255V):

1. It has been easy for me to recognize the target pictogram. [*perception*]
2. Paying attention to the pictograms has caused visual fatigue. [*visual fatigue*]
3. I have found the chosen level of opacity for the pictograms to be comfortable. [*comfort*]
4. The background video has made it difficult for me to perceive the pictograms correctly. [*disturbance*]
5. If I were a patient with severe motor limitations, I would be willing to use this condition for extended use of the interface. [*usability*]

Finally, for the background video tasks in Session 1, aimed at evaluating the impact of stimulus transparency while the user focused on the video and ignored the pictograms, participants responded to the following items after each of the three conditions (A028V, A085V, and A255V):

1. Watching the video with the overlaid pictograms has caused visual fatigue. [*visual fatigue*]
2. The pictograms have made it difficult to perceive the video correctly. [*disturbance*]
3. If I were a patient with severe motor limitations, I would be willing to use this level of transparency for the pictograms to watch a longer video, such as a movie, an episode of a TV series, or the news. [*usability*]

#### Gaze position

To ensure that participants maintained their gaze at the center of the screen (indicated by a cross), a Tobii Eye Tracker 4C (Tobii Technology AB, Stockholm, Sweden) was used during both the BCI tasks and the background video tasks. The device was calibrated for each participant at the beginning of the session. The calculated variables were: (i) the percentage of central fixations (gaze points within 200 px of the screen center, with the pictogram measuring 864 × 864 px), and (ii) the average distance of the gaze position from the center of the pictogram in pixels.

### Statistical analyses

We adopted a model-based statistical approach to account for the distributional characteristics of each dependent variable. Data analyses were conducted in R for BCI accuracy, user target detection, subjective questionnaire items, and eye-tracking measures [34], and in EEGLAB for ERP waveform amplitudes [26]. For the analyses conducted in R, different generalized linear mixed-effects models (GLMMs) were fitted depending on the outcome, using the *glmmTMB* package [35]. BCI accuracy, target detection, and the percentage of central fixations were analyzed with binomial GLMMs, using counts of successes versus failures appropriate to each measure: (i) for BCI accuracy, correct classifications versus misclassifications across 30 trials; (ii) for target detection, detected versus missed target presentations across 300 presentations (30 trials × 10 sequences); and (iii) for percentage of central fixations, samples within the 200-px radius versus samples outside it across all valid eye-tracking samples (the total number of samples varied by participant). Questionnaire responses (Likert scales from 0 to 10) were analyzed with Gaussian LMMs, treating them as approximately continuous, as model residuals conformed to normality. The average distance of gaze position from the pictogram center was modeled with a Gamma GLMM, suitable for positively skewed continuous data. All models included random intercepts for *participant*; fixed effects included *condition*, and for BCI accuracy also *sequence* and the *condition* × *sequence* interaction. Model comparisons were performed with likelihood-ratio tests, and post-hoc pairwise contrasts were derived from estimated marginal means with false discovery rate (FDR) adjustment. ERP analyses in EEGLAB employed permutation-based statistics, with Benjamini–Hochberg correction applied to all multiple comparisons [36].

## Results

In this section, the results obtained from each task designed to investigate the effects of background presence and stimulus transparency are presented. Specifically, the outcomes for each variable studied include: (i) BCI performance, (ii) user target detection, (iii) ERP waveform during the BCI task, (iv) subjective questionnaires, and (v) gaze position measures.

### Background effect

As previously explained, Session 1 examined the background effect using the BCI task with two conditions: A255W with a static white background, and A255V with a video background. The results for each variable related to this effect are detailed below.

#### Brain-computer interface performance

**Fig. 6:**
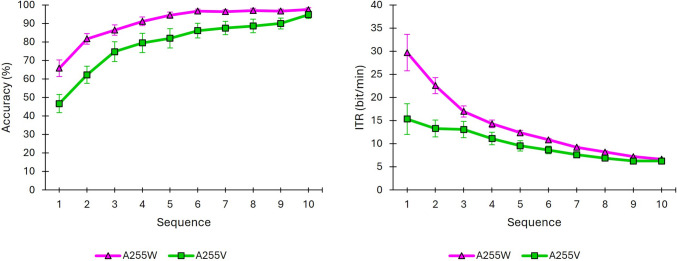
Brain-computer interface performance results (mean ± standard error) for A255W and A255V as a function of the number of sequences elapsed for the variables accuracy (left) and information transfer rate (ITR, right)

A255W and A255V achieved, respectively, an average accuracy of 97.5% and 94.72% by the end of the trial (i.e., 10 sequences) (Fig. 6). These results confirm that the system successfully recognized the target stimulus. However, the ITR reached its minimum value at the end of the trial, indicating that, despite the high accuracy obtained, using such a large number of sequences may not be efficient. For illustrative purposes, accuracy values around 80% can be considered indicative of effective BCI control. Under this reference point, the A255W condition reached an average accuracy of 81.67% in just 2 sequences (22.57 bits/min), whereas the A255V condition required 4 sequences to achieve 79.44% accuracy (11.12 bits/min).

Classification accuracy was analyzed using a binomial GLMM with *condition* (A255W, A255V) and *sequence* (1–10) as fixed factors and *participant* as a random intercept. Likelihood-ratio tests showed that including both *condition* and *sequence* significantly improved model fit relative to simpler models (Δ*χ*2(1) = 216.411, *p* < 0.001 for *condition*, Δ*χ*2(9) = 755.697, *p* < 0.001 for *sequence*). The *condition* × *sequence* interaction was not significant (Δ*χ*2(9) = 8.999, *p* = 0.437), indicating that the effect of *condition* was consistent across repetitions. Post-hoc contrasts revealed significantly lower accuracy in A255V compared to A255W (odds ratio (OR) = 0.331, *p* < 0.001). As expected given the non-significant interaction, this disadvantage of A255V relative to A255W was consistently observed across sequences 1–9 (all *p* < 0.001), but it diminished by sequence 10, where the difference no longer reached significance (*p* = 0.057). Overall, accuracy increased with the number of sequences, approaching ceiling levels after approximately 7–8 sequences in both conditions. Nevertheless, the consistent reduction in accuracy for A255V highlights the detrimental impact of a dynamic video background when stimuli were fully opaque.

#### Target detection

The average percentage of target stimuli correctly detected was 96.8 ± 1.75% in A255W and 95 ± 2.94% in A255V. Detection performance was analyzed using a binomial GLMM, modeling detected versus missed target presentations (300 per condition). Likelihood-ratio tests indicated that including *condition* significantly improved model fit compared to the null model (Δ*χ*2(1) = 14.585, *p* < 0.001). Post-hoc contrasts revealed significantly lower detection accuracy in A255V (mean (M) = 95.6%, standard error (SE) = 0.8%, 95% confidence interval (CI) [93.7–97.0%]) compared to A255W (M = 97.2%, SE = 0.6%, 95% CI [95.9–98.1%]) (OR = 0.632, *p* < 0.001).

#### Event-related potential waveform

**Fig. 7:**
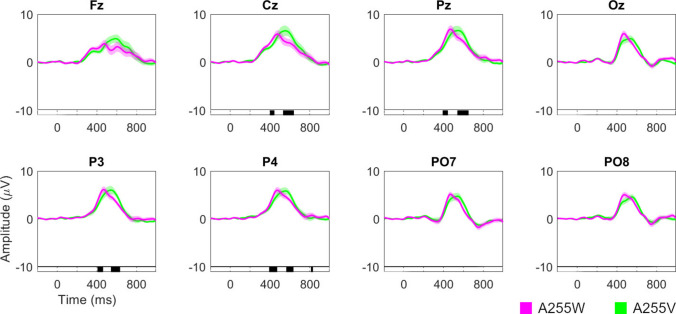
Grand average event-related potential waveforms for the amplitude difference between target and non-target stimuli signals for all channels used (Fz, Cz, Pz, Oz, P3, P4, PO7, and PO8) and for the two conditions evaluating the effect of the presence of a background video (A255W and A255V). Significant intervals are denoted on the bottom line of each plot. The Benjamini–Hochberg procedure was applied to control the false discovery rate and minimize type I errors by adjusting the *p*-value

Figure 7 shows the ERP waveform and the results of statistical analyses comparing the amplitude difference variable (target stimulus amplitude minus non-target stimulus amplitude) between the A255W and A255V conditions. These analyses revealed significant differences in channels Cz, Pz, P3, and P4 within the intervals corresponding to the amplitude peaks of the P300 component for each condition (approximately 380–600 ms). Specifically, the A255V condition exhibited a delay in the P300 component compared to A255W. Therefore, this delay in the ERP is likely attributable to the presence of the background video, an effect that will be further discussed in Section 4.1 Background effect.

#### Subjective items

The subjective items aimed to evaluate the user experience when managing the system under both conditions, with and without a video background. Table 1 presents the average scores for each variable. Self-reported ratings were analyzed with Gaussian LMMs including *condition* as a fixed effect and *participant* as a random intercept. Significant main effects of *condition* were found for perception (*F*(1, 11) = 9, *p* = 0.012) and usability (*F*(1, 11) = 6.807, *p* = 0.024). Specifically, participants reported higher perception and usability in A255W (perception, M = 9.08, SE = 0.4, 95% CI [8.23–9.94]; usability, M = 9, SE = 0.83, 95% CI [7.28–10.72]) compared to A255V (perception, M = 8.33, SE = 4, 95% CI [7.48–9.19]; usability, M = 6.17, SE = 0.83, 95% CI [4.44–7.89]). In contrast, no significant effects of condition were observed for visual fatigue (*F*(1, 11) = 0.164, *p* = 0.693) or comfort (*F*(1, 11) = 4.231, *p* = 0.064).

**Table 1:** Average scores (± standard deviation) for each of the conditions used in the brain-computer interface (BCI) task, either with a white background (A255W) or a video background (A255V)

Item	Condition	
	A255W (1)	A255V (2)
Perception	9.08 ± 1 ^2^	8.33 ± 1.67 ^1^
Visual fatigue	6.33 ± 2.1	6.08 ± 3.09
Comfort	7.83 ± 2.76	6.17 ± 3.07
Usability	9 ± 1.76 ^2^	6.17 ± 3.66 ^1^

Superscripts denote statistically significant differences compared to the indicated condition

#### Gaze position

Regarding the recorded gaze position, the percentage of points recorded within a radius of 200 px from the center of the screen was as follows: A255W, 99.5 ± 0.78%; and A255V, 99.4 ± 0.73%. Similarly, the average distance of the points from the center of the screen was: A255W, 29.1 ± 17.2 px; A255V, 24.3 ± 16.6 px. These results indicate that participants successfully maintained their gaze close to the center of the pictogram (within an 864 px radius). The percentage of gaze points within 200 px was analyzed with a binomial GLMM. Adding *condition* significantly improved model fit compared to the null model (Δ*χ*2(1) = 110.214, *p* < 0.001). Estimated marginal means revealed slightly more stable gaze in A255W (M = 99.79%, SE = 0.09%, 95% CI [99.53–99.91%]) than in A255V (M = 99.73%, SE = 0.12, 95% CI [99.38–99.88%]) (OR = 1.32, *p* < 0.001). However, this effect was not practically meaningful (difference of only 0.06%), and its statistical significance is attributable to the very large number of gaze samples. Consistent with this interpretation, a Gamma GLMM with log link analyzing the average distance from the center found no significant effect of *condition* (Δ*χ*2(1) = 0.558, *p* = 0.455).

### Transparency effect

The transparency effect of the stimuli was examined using three experimental conditions (A255V, A085V, and A028V), in Session 1 with the background video tasks and in Session 2 with the BCI tasks. The results for each variable related to this effect are detailed below.

#### Brain-computer interface performance

**Fig. 8:**
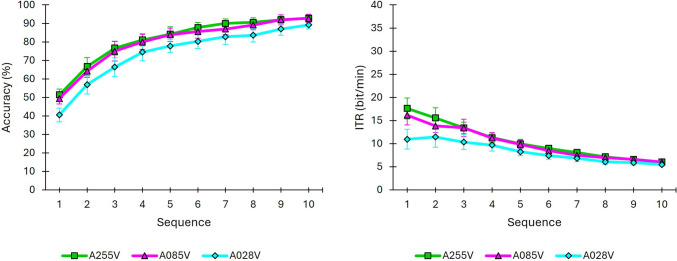
Brain-computer interface performance results (mean ± standard error) for conditions A255V, A085V, and A028V as a function of the number of sequences elapsed for the variables accuracy (left) and information transfer rate (ITR, right)

The average accuracy achieved was 89.17% for A028V, and 92.78% for both A085V and A255V at the end of the trial (i.e., after 10 sequences), indicating that the system successfully discriminated the user’s desired stimulus. Similar to the background effect analysis, the ITR reached its minimum value at the end of the trial, suggesting that, despite the high accuracy obtained, using such a large number of sequences may not be efficient for any of the conditions tested. Individual results per subject and sequence are provided as supplementary material, including both accuracy and ITR. Figure 8 illustrates that the performance between A255V and A085V was quite similar; however, A028V appeared to perform worse throughout the trial compared to the other two conditions.

A binomial GLMM on classification accuracy confirmed that including both *condition* and *sequence* significantly improved model fit relative to models with only one predictor (Δ*χ*2(2) = 81.022, *p* < 0.001 for *condition*; Δ*χ*2(9) = 1178.158, *p* < 0.001 for *sequence*). The *condition* × *sequence* interaction was not significant (Δ*χ*2(18) = 2.711, *p* > 0.999), indicating that the relative differences among conditions remained stable across sequences. Post-hoc contrasts showed that accuracy in A028V was significantly lower than in both A085V (OR = 0.653, *p* < 0.001) and A255V (OR = 0.583, *p* < 0.001), while no significant difference was found between A085V and A255V (OR = 0.894, *p* = 0.091). Sequence-wise comparisons further confirmed the disadvantage of A028V, which yielded lower accuracy across most sequences than both A085V (sequences 1–3, 5, 8 and 9) and A255V (sequences 1–3, 5–9) (all *p* < 0.05).

#### Target detection

Regarding the average percentage of target stimuli detected by the users, the results were as follows: A255V, 94.9 ± 3.13%; A085V, 95 ± 3.77%; A028V, 92.3 ± 4.64%. Detection accuracy was analyzed with a binomial GLMM, modeling detected versus missed target presentations (300 per condition). Including *condition* as a predictor significantly improved model fit compared to the null model (Δ*χ*2(2) = 29.49, *p* < 0.001). Pairwise comparisons revealed that detection was significantly lower in the low-opacity condition (A028V, M = 93.4%, SE = 1.2%, 95% CI [90.6–95.4%]) compared to both the medium-opacity condition (A085V, M = 95.8%, SE = 0.8%, 95% CI [93.8–97.1%]; OR = 0.622, *p* < 0.001) and the high-opacity condition (A255V, M = 95.6%, SE = 0.8%, 95% CI [93.6–97.0%]; OR = 0.644, *p* < 0.001). No significant difference emerged between A085V and A255V (OR = 1.036, *p* = 0.745).

#### Event-related potential waveform

**Fig. 9:**
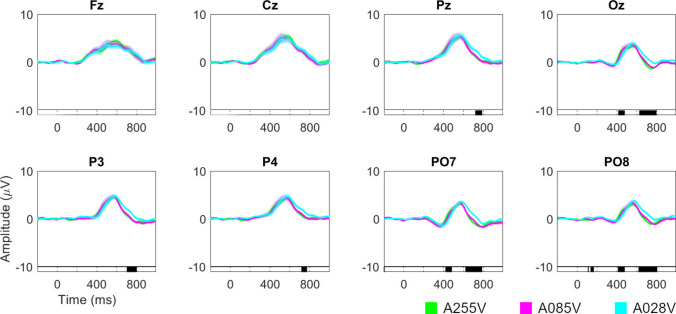
Grand average event-related potential waveforms for the amplitude difference between target and non-target stimuli signals for all channels used (Fz, Cz, Pz, Oz, P3, P4, PO7, and PO8) and for the three conditions varying in the level of stimulus transparency (A255V, A085V, and A028V). Significant intervals are denoted on the bottom line of each plot. The Benjamini–Hochberg procedure was applied to control the false discovery rate and minimize type I errors by adjusting the *p*-value

Figure 9 shows the ERP waveform and the results of a statistical analysis comparing the amplitude of each condition for the target and non-target stimuli, as well as the amplitude differences between them. These analyses showed that the A028V condition (the most transparent) presents a P300 whose amplitude levels take longer to decrease compared to the other two conditions. This effect occurred around 600–800 ms for the channels recorded in the parieto-occipital region (Pz, Oz, P3, P4, PO7, and PO8). Additionally, for the occipital region (Oz, PO7, and PO8), a significant interval was observed around 430 ms during the rise of the P300, where it appears that the most transparent condition takes longer to rise. Therefore, it seems that the level of transparency of the stimuli affects the P300 component. This effect will be discussed in Sect. 4.2 Transparency effect.

#### Subjective items

This subsection presents the scores marked by the users for two tasks: (i) the BCI task of attending to the pictograms presented in Session 2, (ii) the task of attending to the background video, presented in Session 1.

In the BCI task, where participants were instructed to focus on the pictograms while ignoring the background video, several subjective dimensions showed significant differences across conditions (Table 2). For perception, the analysis revealed a robust effect of *condition* (*F*(2, 22) = 15.551, *p* < 0.001). Specifically, the lowest-opacity condition (A028V) received clearly lower ratings (M = 6.17, SE = 0.41, 95% CI [5.33–7.01]) compared to both A085V (M = 8.33, SE = 0.41, 95% CI [7.49–9.17], *p* = 0.009) and A255V (M = 9.08, SE = 0.41, 95% CI [8.24–9.92], *p* = 0.001), whereas no difference was observed between A085V and A255V (*p* = 0.181). In contrast, no significant effect of *condition* was found for visual fatigue (*F*(2, 22) = 0.671, *p* = 0.521). Comfort, however, was strongly influenced by *condition* (*F*(2, 22) = 6, *p* = 0.008), with A028V again producing significantly lower ratings (M = 4.58, SE = 0.72, 95% CI [3.12–6.04]) than both A085V (M = 7.33, SE = 0.72, 95% CI [5.97–8.79], *p* = 0.017) and A255V (M = 7.75, SE = 0.72, 95% CI [6.29–9.21], *p* = 0.013), while no difference emerged between the latter two conditions (*p* = 0.679). A similarly strong pattern was observed for disturbance (*F*(2, 22) = 23.08, *p* < 0.001), with the lowest-opacity condition being rated as substantially more disturbing (A028V: M = 7.83, SE = 0.75, 95% CI [6.27–9.40]) compared to A085V (M = 4.67, SE = 0.75, 95% CI [3.10–6.23], *p* < 0.001) and A255V (M = 3.33, SE = 0.75, 95% CI [1.77–4.90], *p* < 0.001). The difference between A085V and A255V approached but did not reach statistical significance (*p* = 0.063). Finally, usability was also significantly affected by condition (*F*(2, 33) = 4.792, *p* = 0.015), with A028V receiving lower ratings (M = 3.75, SE = 0.72, 95% CI [2.30–5.20]) than both A085V (M = 6.50, SE = 0.72, 95% CI [5.05–7.95], *p* = 0.023) and A255V (M = 6.42, SE = 0.72, 95% CI [4.96–7.87], *p* = 0.023), while the difference between A085V and A255V was negligible (*p* = 0.935). Overall, these findings indicate that the lowest-opacity condition (A028V) was consistently associated with reduced stimulus perception, lower comfort, greater perceived disturbance from the video background, and poorer usability compared with the higher-opacity conditions, which yielded comparable evaluations.

**Table 2:** Average scores (± standard deviation) of the subjective items in the brain-computer interface (BCI) task, where participants attended to the pictograms and ignored the background video, across the conditions with a video background and varying levels of stimulus transparency (A255V, A085V, and A028V)

Item	Condition		
	A255V (1)	A085V (2)	A028V (3)
Perception	9.08 ± 1 ^3^	8.33 ± 1.16 ^3^	6.17 ± 1.95 ^1,2^
Visual fatigue	6.58 ± 1.88	5.58 ± 2.75	6.17 ± 2.59
Comfort	7.75 ± 1.76 ^3^	7.33 ± 2.15 ^3^	4.58 ± 3.29 ^1,2^
Disturbance	3.33 ± 2.67 ^3^	4.67 ± 2.5 ^3^	7.83 ± 2.62 ^1,2^
Usability	6.42 ± 2.23 ^3^	6.5 ± 2.15 ^3^	3.75 ± 2.96 ^1,2^

Superscripts denote statistically significant differences compared to the indicated condition

Turning to the video task, in which participants were instructed to focus on the background video while ignoring the pictograms, transparency strongly modulated all subjective dimensions (Table 3). For visual fatigue, the effect of *condition* was highly significant (*F*(2, 22) = 29.87, *p* < 0.001). Specifically, ratings for the lowest-opacity condition (A028V: M = 1.25, SE = 0.60, 95% CI [0–2.5]) were significantly lower than for both A085V (M = 3.33, SE = 0.6, 95% CI [2.09–4.58], *p* = 0.004) and A255V (M = 6.17, SE = 0.6, 95% CI [4.92–7.41], *p* < 0.001), and A085V was in turn rated lower than A255V (*p* < 0.001). A similar pattern was observed for perceived disturbance (*F*(2, 22) = 29.04, *p* < 0.001), which decreased progressively with increasing stimulus transparency. The lowest-opacity condition (A028V: M = 1.67, SE = 0.71, 95% CI [0.18–3.15]) was rated significantly lower than both A085V (M = 4, SE = 0.71, 95% CI [2.52–5.49], *p* = 0.001) and A255V (M = 6.42, SE = 0.71, 95% CI [4.93–7.9], *p* < 0.001), while A085V also received lower ratings than A255V (*p* = 0.001). Finally, as expected when participants were asked to watch the background video, usability increased as stimulus transparency rose (*F*(2, 22) = 22.14, *p* < 0.001). In this case, A028V was rated highest (M = 8, SE = 0.65, 95% CI [6.66–9.34]), significantly above A085V (M = 6.42, SE = 0.65, 95% CI [5.07–7.76], *p* = 0.034) and A255V (M = 3.42, SE = 0.65, 95% CI [2.07–4.76], *p* < 0.001), with A085V again rated higher than A255V (*p* < 0.001).

**Table 3:** Average scores (± standard deviation) of the subjective items in the background video task, where participants attended to the background video and ignored the pictograms, across the conditions with a video background and varying levels of pictogram transparency (A255V, A085V, and A028V)

Item	Condition		
	A255V (1)	A085V (2)	A028V (3)
Visual fatigue	6.17 ± 2.44 ^2,3^	3.33 ± 2.27 ^1,3^	1.25 ± 1.42 ^1,2^
Disturbance	6.42 ± 3.09 ^2,3^	4 ± 2.49 ^1,3^	1.67 ± 1.56 ^1,2^
Usability	3.42 ± 2.84 ^2,3^	6.42 ± 1.78 ^1,3^	8 ± 2 ^1,2^

Superscripts denote statistically significant differences compared to the indicated condition

#### Gaze position

In the BCI task, the percentage of points recorded within a 200-pixel radius from the center of the screen were as follows: A255V, 98.1 ± 4.61%; A085V, 98.5 ± 2.06%; and A028V, 98.3 ± 2.27%. Additionally, the average distance of these points from the center of the screen was: A255V, 43.1 ± 35.4 pixels; A085V, 44.2 ± 31.2 pixels; and A028V, 52.5 ± 32 pixels. These results indicate that users effectively maintained their gaze within the central area of the pictogram (864-pixel radius). The percentage of gaze points within 200 pixels of the screen center was analyzed with a binomial GLMM. The model including *condition* provided a significantly better fit than the null model (Δ*χ*2(2) = 391.624, *p* < 0.001). Pairwise contrasts showed that gaze stability in A255V (M = 99.3%, SE = 0.32%, 95% CI [98.27–99.72%]) was slightly but significantly lower than in both A028V (M = 99.45%, SE = 0.26%, 95% CI [98.64–99.78%], *p* < 0.001) and A085V (M = 99.46%, SE = 0.25%, 95% CI [98.66–99.78%], *p* < 0.001). Nonetheless, the absolute differences between conditions were negligible (≤ 0.16%), indicating that these effects reached significance mainly due to the very large number of gaze samples included in the model. Consistent with this interpretation, a Gamma GLMM with log link analyzing average distance from the pictogram center found no significant effect of condition (Δ*χ*2(2) = 2.376, *p* = 0.305).

In the background video task, the percentage of points recorded within a 200-pixel radius from the center of the screen were: A255V, 91.5 ± 18.4%; A085V, 93 ± 11%; and A028V, 92.2 ± 15.1%. Similarly, the average distance of these points from the center of the screen was: A255V, 63.6 ± 84.6 pixels; A085V, 63.1 ± 60 pixels; and A028V, 60.3 ± 69.1 pixels. These results confirm that users maintained their gaze within the central area of the pictogram they were instructed to ignore (864-pixel radius). The binomial GLMM indicated that including *condition* improved model fit compared to the null model (Δ*χ*2(2) = 381.57, *p* < 0.001). Pairwise contrasts revealed statistically significant differences among all conditions (all *p* < 0.001), with A085V (M = 98.2%, 95% CI [94.8–99.4]) showing slightly higher stability than A028V (M = 97.9%, SE = 1.14%, 95% CI [94.0–99.3]), and both A028V and A085V outperforming A255V (M = 97.6%, 95% CI [93.3–99.2]). However, as in the BCI task, the magnitude of these differences was very small (< 0.6%), suggesting that they are unlikely to reflect practically meaningful variations in gaze stability but rather the high sensitivity of the binomial model given the large sample size. Corroborating this point, a Gamma GLMM analyzing average distance from the pictogram center showed no significant effect of *condition* (Δ*χ*2(2) = 2.776, *p* = 0.25).

## Discussion

In this section, we will discuss and contextualize the results of the present study in relation to the relevant literature. This discussion is organized into three parts: (i) 4.1 Background effect, where the impact of the video background on the performance will be examined; (ii) 4.2 Transparency effect, which addresses the influence of stimuli (i.e., pictograms) transparency; and (iii) 4.3 Limitations and future work, which outlines the study’s limitations and proposes potential solutions for future research.

### Background effect

The results concerning the background effect on visual ERP-BCI systems using RSVP are clear: incorporating a video background—specifically, a dynamic background with audio—has a detrimental impact on BCI performance, user target detection, and subjective experience (including perception and usability). Additionally, it leads to an increase in the latency of the P300 component.

Regarding BCI performance, this study corroborates the findings of Kim et al. [20], who observed negative effects of a video background under a single-character paradigm (specifically, using four stimuli, each positioned in a corner of the screen). This study extends their results to RSVP, demonstrating similar detrimental effects on performance with a larger number of commands, trials, and participants. In contrast, Pitt et al. [37] found no significant performance differences between ERP-BCI conditions with a complex but static background and a homogeneous white background. Therefore, future research should consider that a complex, dynamic background with audio may be a critical factor negatively impacting BCI performance.

The reduced performance observed under the video background condition may stem from several interrelated factors. First, the lower detection rates of target stimuli in the video background condition (A255W, 96.8%; A255V, 95%) could negatively impact performance, as users who do not perceive the target stimulus will not trigger the BCI’s detection capabilities based on the EEG signal. This objective finding aligns with users’ subjective reports of greater difficulty in perceiving target stimuli (A255W, 9.08 points; A255V, 8.33 points) [17]. Second, the increased difficulty users experience when performing the task with a video background may require more cognitive resources, which could impair ERP-BCI performance [38]. Additionally, as indicated by Polich [39], P300 latency has been commonly used as a metric for timing mental events, suggesting that increased latency for A255V might result from delayed recognition of the target stimulus due to its greater difficulty. Third, another source could be the impact of background heterogeneity across different trials on the ERPs. In our paradigm, the video content during the ISI changes in lighting, colors, and movement, which can introduce variability into the ERP waveform associated with both target and non-target stimuli [40, 41]. This dynamic background variability could add noise to the classifier’s model, making it more challenging to distinguish between target and non-target stimuli.

### Transparency effect

The results concerning the impact of stimulus transparency levels on performance indicated a significant decrease for condition A028V, with no significant differences observed between conditions A255V and A085V. Therefore, in terms of performance, condition A085V appears to be as effective as A255V when using standard opaque pictograms. These findings are consistent with those of Li et al. [21] and Lian et al. [22], who demonstrated that excessive reduction in luminance contrast can negatively affect BCI performance. Regarding target detection, the most transparent condition, A028V (92.3%), yielded significantly lower accuracy compared to A085V (95.4%) and A255V (94.9%). Therefore, as noted in the background effect discussion, one possible explanation for the poorer BCI performance in A028V could be its lower target detection.

In terms of ERP waveform, Lian et al. [22] reported a decrease in P300 amplitude and an increase in its peak latency for the condition with the lowest luminance contrast, while Li et al. [21] observed similar trends but without significant differences. In our study, no decrease in amplitude or peak delay of the P300 was observed, although a delay in the P300’s decline was noted for the condition with the most transparent stimuli (A028V). This discrepancy might be due to our transparency level not being high enough or due to specific aspects of our paradigm, such as the type of background or the use of pictograms as stimuli.

Regarding subjective measures, when users were tasked with selecting a pictogram (i.e., the BCI task), the results were similar across ease of perception, comfort, video disturbance, and usability: the lowest-opacity condition (A028V) yielded the poorest ratings on all these dimensions, whereas no significant differences were found between the other two conditions (Table 2). These findings, similar to the background effect results, suggest that the A028V condition may impose a higher mental load on users, which could contribute to the observed performance discrepancies [38]. On the other hand, for the task of attending to the background video (i.e., background video task), higher transparency of the stimuli led to better ratings across all three variables (visual fatigue, disturbance from stimuli, and usability) (Table 3). Notably, users rated usability significantly lower for A028V in the BCI task (3.75 points) compared to A255V (6.42 points), and lower for A255V in the background video task (3.42 points) compared to A028V (8 points). Users thus expressed dissatisfaction with the most extreme transparency conditions for either task. As anticipated, recommending an appropriate level of transparency should be task-dependent. While it was expected that users would prefer opaque conditions for the BCI task and transparent conditions for watching the video, these results emphasize the need to balance stimulus transparency levels effectively.

### Limitations and future work

The present study has certain limitations that should be considered, either to clarify the impact of our findings or to be resolved in future studies.

Firstly, as described in previous works, the need to validate results in the clinical population remains a challenge in the BCI field [42]. In the present study, most of the participants were young adult college students with no reports of neurological problems. This population differed substantially from the main target populations of gaze-independent ERP-BCI, who are middle-aged or elderly patients with severe motor problems [43]. However, not all BCI applications are intended for patients; some are aimed at a non-clinical population (e.g., AR applications for entertainment [44]), which could benefit from the findings presented here.

Secondly, although each experimental session lasted around 105 min, the effective use of the BCI per experimental condition did not exceed 20 min. This limitation was due to the need to manage multiple experimental conditions during each session, as well as the time required for explaining the procedures, setting up the EEG equipment, and completing questionnaires. Such a limited duration may be insufficient for a comprehensive assessment of system performance and user experience, particularly given that most participants in this study had no prior experience with BCI applications. Unlike patients or frequent users who might be more accustomed to prolonged use of such interfaces, our participants were less familiar with the technology. In real-world applications, ERP-BCIs may be used for extended periods of uninterrupted interaction or across repeated daily sessions. Both scenarios can introduce cumulative effects, such as fatigue or training, which may in turn influence performance, attention, and overall user acceptance of the technology. Future research should therefore assess ERP-BCIs under longer and repeated usage conditions in order to better capture their robustness, usability, and long-term impact on the user experience.

Thirdly, although this study addresses practical implications for integrating BCI systems into real-world environments—such as for home automation, watching movies, or navigating the internet—it was conducted in a controlled laboratory setting. This environment differs significantly from the real-world scenarios in which users would typically interact with these systems. Participants performed the tasks in a distraction-free room, following a protocol designed to theoretically evaluate the impact of (i) a dynamic background with audio and (ii) stimuli transparency. However, this controlled setting does not account for variables present in real-world use, such as variations in ambient light, auditory distractions [38], or the use of lower-quality commercial EEG systems [45]. Future research should consider evaluating BCI performance in more varied and realistic settings to better understand how these factors might influence user experience and system effectiveness.

Fourthly, building on the previous point, future studies should enhance the paradigm presented here by incorporating additional functionalities. These could include asynchronous online control [46], integrating BCI systems for controlling applications or devices in the user’s environment [13], or dynamically adjusting the alpha channel based on the background. Previous research has demonstrated that factors such as the distance between the user’s gaze position and the target stimulus, or the size of the stimuli, can both positively and negatively affect ERP-BCI performance in gaze-independent BCIs [33, 47]. While increased stimulus salience generally enhances system performance, it is crucial to strike a balance, as excessive salience can cause greater discomfort when users need to ignore the stimuli and focus on the background. Future research should investigate how these variables interact to optimize both the performance and usability of BCI systems.

Finally, our findings have direct implications for the development of real-time ERP-based BCIs aimed at improving the quality of life of individuals with severe motor impairments. As outlined in the introduction, semi-transparent stimuli presented over dynamic backgrounds are particularly relevant in scenarios where users must divide attention between the interface and ongoing audiovisual content. Such systems could operate unobtrusively in the background and be activated on demand—for instance, to issue simple commands while watching a movie (e.g., pause or adjust the volume), to select options during a video call, or to communicate basic needs through an interface running continuously on AR glasses (e.g., “I need help” or “I want to watch television”). Future work should therefore focus on implementing and validating these real-time applications with the target end-user populations under extended and repeated use conditions, while integrating the additional functionalities previously discussed.

## Conclusions

To our knowledge, this study is the first to assess the effects of background video and stimulus transparency on ERP-BCI performance under RSVP. The findings are significant for future research aiming to integrate these systems into real or virtual user environments. On one hand, this study demonstrates that a dynamic audiovisual background negatively impacts system performance. This suggests that the background effect is not confined to the single-character paradigm [20] but may be a general issue for visual ERP-BCIs. Despite the negative impact of the video background, users achieved high accuracy, indicating that while the background effect presents challenges, it does not preclude effective performance. Future research could explore integrating these systems into practical applications, such as television, home automation or AR applications, or developing strategies to mitigate the impact of the background on performance. On the other hand, stimulus transparency also influenced ERP-BCI usability, depending on the user’s task. As hypothesized based on prior literature without a video background, performance decreased with increasing stimulus transparency [21, 22]. However, the results suggest that an intermediate level of transparency (A085V) does not significantly differ in performance from the benchmark condition (A255V) for communication tasks (BCI task) but performs better for tasks focused on background perception (background video task). Since all conditions achieved high accuracy by the end of the trial, the optimal level of transparency should be tailored to the specific application and user preferences.

In summary, this study highlights the impact of two critical variables for integrating ERP-BCIs under RSVP into user environments: the background, and stimulus transparency. These findings underscore new challenges for the future of BCIs and their potential uses, such as video players, web browsers or AR-based applications. Additionally, implementing the discussed functionalities is strongly recommended to develop applications that can improve the quality of life for patients with severe motor impairments.

## Supplementary Information

Below is the link to the electronic supplementary material.

Supplementary file1 (PDF 1082 KB)

## Data Availability

The datasets generated for this study are available at 10.17605/OSF.IO/C6HKM.
