# Supplementary material for: Evaluation of video background and stimulus transparency in a visual ERP-based BCI under RSVP
Source: Med Biol Eng Comput. 2026 Jan 10;64(3):1065–82. doi: 10.1007/s11517-025-03498-5 (PMC13061822; doi:10.1007/s11517-025-03498-5)
Supplement: Supplementary file 1 — Supplementary file1 (PDF 1082 KB) [file 11517_2025_3498_MOESM1_ESM.pdf]

Evaluation of video background and stimulus transparency in a visual ERP-based BCI under RSVP

Supplementary material

Tables 1-4 present the results for each participant—both accuracy (%) and information transfer rate (ITR, bit/min)—for each sequence in the brain-computer interface (BCI) task of Session 1, using the A255W and A255V conditions.

Tables 5-10 show the results for each participant—both accuracy (%) and information transfer rate (ITR, bit/min)—for each sequence in the BCI task of Session 2, using the A255V, A085V, and A028V conditions.

Figures 1 and 2 correspond to the grand average of the event-related potential waveforms for the target and non-target stimuli during the BCI tasks of Session 1 and Session 2 for each of the conditions.

Table 1. Accuracy (%) results in the brain-computer interface (BCI) task of Session 1 for each participant and each sequence under the A255W condition (opaque pictograms with a static white background).

User	Sequence									
	1	2	3	4	5	6	7	8	9	10
BU01	90.00	93.33	100.00	100.00	100.00	100.00	100.00	100.00	100.00	100.00
BU02	66.67	80.00	76.67	83.33	90.00	93.33	93.33	93.33	96.67	96.67
BU03	60.00	83.33	90.00	93.33	93.33	96.67	96.67	96.67	96.67	100.00
BU04	70.00	83.33	86.67	96.67	100.00	96.67	90.00	100.00	96.67	96.67
BU05	40.00	70.00	83.33	86.67	90.00	96.67	96.67	96.67	96.67	100.00
BU06	56.67	66.67	70.00	76.67	90.00	93.33	93.33	96.67	96.67	96.67
BU07	40.00	76.67	76.67	83.33	83.33	90.00	93.33	86.67	86.67	90.00
BU08	73.33	86.67	93.33	96.67	100.00	100.00	100.00	100.00	96.67	96.67
BU09	80.00	100.00	96.67	100.00	100.00	100.00	100.00	100.00	100.00	100.00
BU10	60.00	73.33	76.67	83.33	90.00	93.33	93.33	93.33	93.33	93.33
BU11	70.00	76.67	90.00	93.33	96.67	100.00	100.00	100.00	100.00	100.00
BU12	83.33	90.00	96.67	100.00	100.00	100.00	100.00	100.00	100.00	100.00
Mean	65.83	81.67	86.39	91.11	94.44	96.67	96.39	96.94	96.67	97.50
SD	15.58	9.80	9.69	8.08	5.74	3.48	3.61	4.13	3.76	3.22

Table 2. Accuracy (%) results in the BCI task of Session 1 for each participant and each sequence under the A255V condition (opaque pictograms with a background video).

User	Sequence									
	1	2	3	4	5	6	7	8	9	10
BU01	73.33	83.33	93.33	96.67	100.00	100.00	100.00	100.00	100.00	100.00
BU02	56.67	76.67	86.67	100.00	100.00	100.00	96.67	100.00	100.00	100.00
BU03	23.33	40.00	43.33	53.33	66.67	63.33	70.00	70.00	76.67	86.67
BU04	43.33	60.00	60.00	63.33	70.00	70.00	73.33	80.00	80.00	86.67
BU05	43.33	63.33	90.00	93.33	96.67	96.67	96.67	93.33	90.00	93.33
BU06	43.33	63.33	76.67	83.33	86.67	86.67	93.33	96.67	100.00	100.00
BU07	23.33	53.33	66.67	70.00	70.00	73.33	66.67	66.67	76.67	80.00
BU08	46.67	60.00	80.00	86.67	83.33	93.33	96.67	100.00	96.67	100.00
BU09	73.33	83.33	96.67	93.33	100.00	100.00	100.00	100.00	100.00	100.00
BU10	30.00	30.00	46.67	43.33	40.00	70.00	76.67	76.67	76.67	90.00
BU11	40.00	63.33	63.33	80.00	80.00	83.33	83.33	83.33	86.67	100.00
BU12	63.33	70.00	93.33	90.00	90.00	96.67	96.67	96.67	96.67	100.00
Mean	46.67	62.22	74.72	79.44	81.94	86.11	87.50	88.61	90.00	94.72
SD	17.17	15.91	18.50	18.14	18.00	13.69	12.64	12.59	10.15	7.17

Table 3. Information transfer rate (ITR, bit/min) results in the BCI task of Session 1 for each participant and each sequence under the A255W condition (opaque pictograms with a static white background).

User	Sequence									
	1	2	3	4	5	6	7	8	9	10
BU01	54.34	29.54	23.73	17.79	14.24	11.86	10.17	8.90	7.91	7.12
BU02	28.86	21.06	12.85	11.48	10.87	9.85	8.44	7.39	7.16	6.44
BU03	23.21	22.97	18.11	14.77	11.82	10.73	9.20	8.05	7.16	7.12
BU04	31.92	22.97	16.66	16.10	14.24	10.73	7.76	8.90	7.16	6.44
BU05	9.62	15.96	15.31	12.50	10.87	10.73	9.20	8.05	7.16	7.12
BU06	20.60	14.43	10.64	9.63	10.87	9.85	8.44	8.05	7.16	6.44
BU07	9.62	19.27	12.85	11.48	9.19	9.06	8.44	6.25	5.55	5.43
BU08	35.14	24.99	19.69	16.10	14.24	11.86	10.17	8.90	7.16	6.44
BU09	42.13	35.59	21.47	17.79	14.24	11.86	10.17	8.90	7.91	7.12
BU10	23.21	17.57	12.85	11.48	10.87	9.85	8.44	7.39	6.56	5.91
BU11	31.92	19.27	18.11	14.77	12.88	11.86	10.17	8.90	7.91	7.12
BU12	45.93	27.17	21.47	17.79	14.24	11.86	10.17	8.90	7.91	7.12
Mean	29.71	22.57	16.98	14.31	12.38	10.84	9.23	8.21	7.22	6.65
SD	13.61	6.06	4.16	2.90	1.84	1.02	0.91	0.86	0.68	0.57

Table 4. Information transfer rate (ITR, bit/min) results in the BCI task of Session 1 for each participant and each sequence under the A255V condition (opaque pictograms with a background video).

User	Sequence									
	1	2	3	4	5	6	7	8	9	10
BU01	35.14	22.97	19.69	16.10	14.24	11.86	10.17	8.90	7.91	7.12
BU02	20.60	19.27	16.66	17.79	14.24	11.86	9.20	8.90	7.91	7.12
BU03	2.31	4.81	3.85	4.53	5.77	4.33	4.56	3.99	4.28	5.00
BU04	11.54	11.60	7.74	6.49	6.38	5.32	5.02	5.27	4.68	5.00
BU05	11.54	12.98	18.11	14.77	12.88	10.73	9.20	7.39	6.04	5.91
BU06	11.54	12.98	12.85	11.48	10.00	8.33	8.44	8.05	7.91	7.12
BU07	2.31	9.06	9.62	7.98	6.38	5.86	4.12	3.61	4.28	4.21
BU08	13.60	11.60	14.04	12.50	9.19	9.85	9.20	8.90	7.16	7.12
BU09	35.14	22.97	21.47	14.77	14.24	11.86	10.17	8.90	7.91	7.12
BU10	4.75	2.38	4.53	2.88	1.92	5.32	5.51	4.82	4.28	5.43
BU11	9.62	12.98	8.65	10.53	8.43	7.66	6.56	5.74	5.55	7.12
BU12	25.96	15.96	19.69	13.59	10.87	10.73	9.20	8.05	7.16	7.12
Mean	15.34	13.30	13.08	11.12	9.54	8.64	7.61	6.87	6.26	6.28
SD	11.50	6.36	6.15	4.74	3.96	2.87	2.29	2.06	1.57	1.10

Table 5. Accuracy (%) results in the BCI task of Session 2 for each participant and each sequence under the A255V condition (opaque pictograms with a background video).

User	Sequence									
	1	2	3	4	5	6	7	8	9	10
TU01	53.33	70.00	73.33	73.33	83.33	86.67	83.33	93.33	93.33	100.00
TU02	53.33	63.33	66.67	70.00	76.67	76.67	76.67	76.67	83.33	83.33
TU03	30.00	50.00	76.67	66.67	60.00	80.00	83.33	76.67	73.33	76.67
TU04	73.33	90.00	93.33	90.00	96.67	100.00	100.00	100.00	100.00	100.00
TU05	43.33	53.33	66.67	76.67	83.33	80.00	93.33	96.67	93.33	93.33
TU07	60.00	73.33	90.00	93.33	100.00	100.00	100.00	100.00	100.00	100.00
TU08	33.33	26.67	36.67	50.00	46.67	56.67	60.00	60.00	70.00	66.67
TU09	43.33	60.00	80.00	90.00	86.67	90.00	90.00	93.33	93.33	96.67
TU10	53.33	60.00	76.67	83.33	83.33	86.67	100.00	96.67	100.00	100.00
TU11	53.33	80.00	86.67	96.67	96.67	96.67	96.67	96.67	96.67	96.67
TU12	63.33	90.00	86.67	96.67	96.67	100.00	100.00	100.00	100.00	100.00
TU13	56.67	83.33	86.67	86.67	100.00	100.00	96.67	96.67	100.00	100.00
Mean	51.39	66.67	76.67	81.11	84.17	87.78	90.00	90.56	91.94	92.78
SD	12.26	18.48	15.31	14.17	16.58	13.13	12.31	12.62	10.68	11.18

Table 6. Accuracy (%) results in the BCI task of Session 2 for each participant and each sequence under the A085V condition (medium transparency pictograms with a background video).

User	Sequence									
	1	2	3	4	5	6	7	8	9	10
TU01	40.00	60.00	63.33	70.00	73.33	93.33	86.67	93.33	93.33	96.67
TU02	46.67	56.67	73.33	70.00	73.33	70.00	83.33	80.00	93.33	96.67
TU03	40.00	60.00	66.67	63.33	70.00	80.00	80.00	80.00	90.00	90.00
TU04	70.00	83.33	100.00	100.00	100.00	100.00	100.00	100.00	100.00	100.00
TU05	40.00	50.00	56.67	63.33	73.33	70.00	66.67	73.33	76.67	76.67
TU07	53.33	73.33	93.33	100.00	100.00	96.67	100.00	100.00	100.00	100.00
TU08	30.00	43.33	33.33	53.33	56.67	53.33	56.67	66.67	60.00	66.67
TU09	50.00	80.00	100.00	93.33	93.33	93.33	96.67	100.00	100.00	100.00
TU10	53.33	50.00	66.67	76.67	83.33	83.33	90.00	83.33	93.33	90.00
TU11	53.33	66.67	70.00	83.33	90.00	96.67	93.33	100.00	100.00	100.00
TU12	50.00	73.33	86.67	90.00	93.33	93.33	90.00	93.33	96.67	96.67
TU13	66.67	73.33	90.00	96.67	100.00	96.67	100.00	100.00	100.00	100.00
Mean	49.44	64.17	75.00	80.00	83.89	85.56	86.94	89.17	91.94	92.78
SD	11.36	12.80	19.82	16.08	14.34	14.52	13.67	11.99	12.10	10.72

Table 7. Accuracy (%) results in the BCI task of Session 2 for each participant and each sequence under the A028V condition (high transparency pictograms with a background video).

User	Sequence									
	1	2	3	4	5	6	7	8	9	10
TU01	33.33	43.33	46.67	46.67	70.00	70.00	73.33	70.00	70.00	73.33
TU02	23.33	50.00	63.33	80.00	76.67	80.00	80.00	93.33	96.67	93.33
TU03	20.00	43.33	56.67	63.33	63.33	63.33	66.67	70.00	76.67	83.33
TU04	53.33	86.67	93.33	96.67	96.67	96.67	96.67	96.67	96.67	96.67
TU05	36.67	46.67	63.33	73.33	76.67	76.67	86.67	76.67	86.67	93.33
TU07	63.33	83.33	83.33	90.00	90.00	100.00	100.00	100.00	100.00	100.00
TU08	36.67	40.00	50.00	63.33	53.33	56.67	53.33	60.00	66.67	76.67
TU09	43.33	50.00	60.00	76.67	83.33	86.67	90.00	90.00	96.67	96.67
TU10	33.33	30.00	30.00	40.00	60.00	60.00	60.00	60.00	60.00	66.67
TU11	36.67	63.33	86.67	83.33	86.67	90.00	93.33	96.67	96.67	96.67
TU12	53.33	76.67	83.33	83.33	86.67	90.00	96.67	93.33	100.00	96.67
TU13	53.33	70.00	80.00	96.67	90.00	93.33	96.67	96.67	96.67	96.67
Mean	40.56	56.94	66.39	74.44	77.78	80.28	82.78	83.61	86.94	89.17
SD	13.09	18.50	19.15	18.16	13.58	14.87	15.94	15.21	14.60	11.20

Table 8. Information transfer rate (ITR, bit/min) results in the BCI task of Session 2 for each participant and each sequence under the A255V condition (opaque pictograms with a background video).

User	Sequences									
	1	2	3	4	5	6	7	8	9	10
TU01	18.13	15.96	11.71	8.79	9.19	8.33	6.56	7.39	6.56	7.12
TU02	18.13	12.98	9.62	7.98	7.71	6.42	5.51	4.82	5.10	4.59
TU03	4.75	7.90	12.85	7.22	4.64	7.02	6.56	4.82	3.90	3.85
TU04	35.14	27.17	19.69	13.59	12.88	11.86	10.17	8.90	7.91	7.12
TU05	11.54	9.06	9.62	9.63	9.19	7.02	8.44	8.05	6.56	5.91
TU07	23.21	17.57	18.11	14.77	14.24	11.86	10.17	8.90	7.91	7.12
TU08	6.22	1.72	2.62	3.95	2.72	3.43	3.32	2.90	3.55	2.89
TU09	11.54	11.60	14.04	13.59	10.00	9.06	7.76	7.39	6.56	6.44
TU10	18.13	11.60	12.85	11.48	9.19	8.33	10.17	8.05	7.91	7.12
TU11	18.13	21.06	16.66	16.10	12.88	10.73	9.20	8.05	7.16	6.44
TU12	25.96	27.17	16.66	16.10	12.88	11.86	10.17	8.90	7.91	7.12
TU13	20.60	22.97	16.66	12.50	14.24	11.86	9.20	8.05	7.91	7.12
Mean	17.62	15.56	13.43	11.31	9.98	8.98	8.10	7.18	6.58	6.07
SD	8.46	7.92	4.70	3.82	3.69	2.73	2.22	1.94	1.59	1.48

Table 9. Information transfer rate (ITR, bit/min) results in the BCI task of Session 2 for each participant and each sequence under the A085V condition (medium transparency pictograms with a background video).

User	Sequences									
	1	2	3	4	5	6	7	8	9	10
TU01	9.62	11.60	8.65	7.98	7.03	9.85	7.14	7.39	6.56	6.44
TU02	13.60	10.30	11.71	7.98	7.03	5.32	6.56	5.27	6.56	6.44
TU03	9.62	11.60	9.62	6.49	6.38	7.02	6.02	5.27	6.04	5.43
TU04	31.92	22.97	23.73	17.79	14.24	11.86	10.17	8.90	7.91	7.12
TU05	9.62	7.90	6.87	6.49	7.03	5.32	4.12	4.39	4.28	3.85
TU07	18.13	17.57	19.69	17.79	14.24	10.73	10.17	8.90	7.91	7.12
TU08	4.75	5.77	2.07	4.53	4.12	3.02	2.94	3.61	2.58	2.89
TU09	15.79	21.06	23.73	14.77	11.82	9.85	9.20	8.90	7.91	7.12
TU10	18.13	7.90	9.62	9.63	9.19	7.66	7.76	5.74	6.56	5.43
TU11	18.13	14.43	10.64	11.48	10.87	10.73	8.44	8.90	7.91	7.12
TU12	15.79	17.57	16.66	13.59	11.82	9.85	7.76	7.39	7.16	6.44
TU13	28.86	17.57	18.11	16.10	14.24	10.73	10.17	8.90	7.91	7.12
Mean	16.16	13.85	13.43	11.22	9.83	8.50	7.54	6.96	6.61	6.04
SD	7.90	5.53	6.86	4.69	3.51	2.78	2.35	2.00	1.67	1.41

Table 10. Information transfer rate (ITR, bit/min) results in the BCI task of Session 2 for each participant and each sequence under the A028V condition (high transparency pictograms with a background video).

User	Sequences									
	1	2	3	4	5	6	7	8	9	10
TU01	6.22	5.77	4.53	3.40	6.38	5.32	5.02	3.99	3.55	3.51
TU02	2.31	7.90	8.65	10.53	7.71	7.02	6.02	7.39	7.16	5.91
TU03	1.37	5.77	6.87	6.49	5.19	4.33	4.12	3.99	4.28	4.59
TU04	18.13	24.99	19.69	16.10	12.88	10.73	9.20	8.05	7.16	6.44
TU05	7.85	6.80	8.65	8.79	7.71	6.42	7.14	4.82	5.55	5.91
TU07	25.96	22.97	15.31	13.59	10.87	11.86	10.17	8.90	7.91	7.12
TU08	7.85	4.81	5.26	6.49	3.63	3.43	2.59	2.90	3.21	3.85
TU09	11.54	7.90	7.74	9.63	9.19	8.33	7.76	6.79	7.16	6.44
TU10	6.22	2.38	1.58	2.41	4.64	3.87	3.32	2.90	2.58	2.89
TU11	7.85	12.98	16.66	11.48	10.00	9.06	8.44	8.05	7.16	6.44
TU12	18.13	19.27	15.31	11.48	10.00	9.06	9.20	7.39	7.91	6.44
TU13	18.13	15.96	14.04	16.10	10.87	9.85	9.20	8.05	7.16	6.44
Mean	10.96	11.46	10.36	9.71	8.25	7.44	6.85	6.10	5.90	5.50
SD	7.51	7.61	5.65	4.46	2.86	2.80	2.58	2.22	1.97	1.40

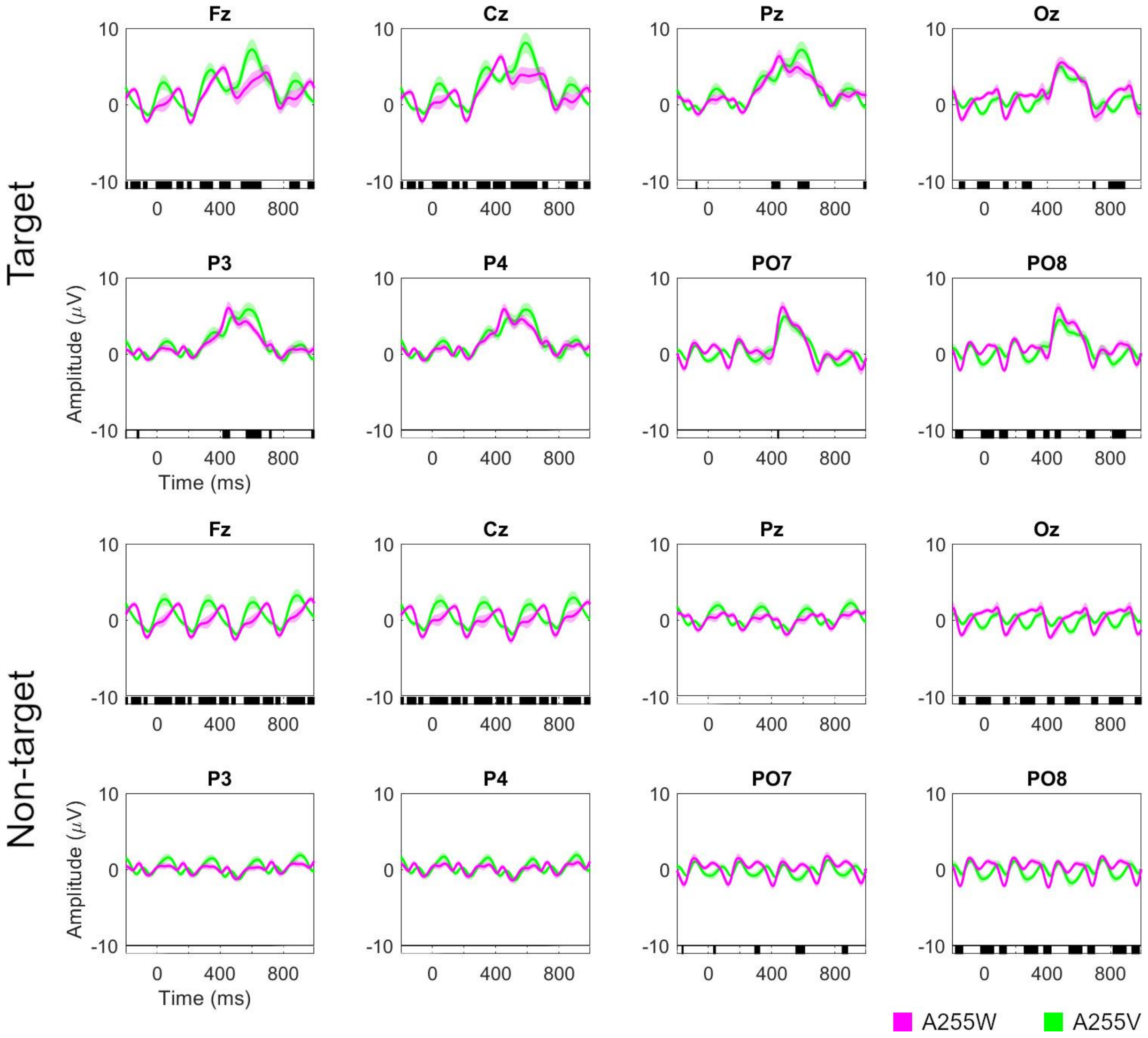

Figure 1. Grand average event-related potential waveforms for the target and non-target stimuli signals for all channels used (Fz, Cz, Pz, Oz, P3, P4, PO7, and PO8) and for the two conditions evaluating the effect of the presence of a background video in the brain-computer interface (BCI) task of Session 1 (A255W and A255V). Significant intervals are denoted on the bottom line of each plot. The Benjamini-Hochberg procedure was applied to control the false discovery rate and minimize type I errors by adjusting the *p*-value.

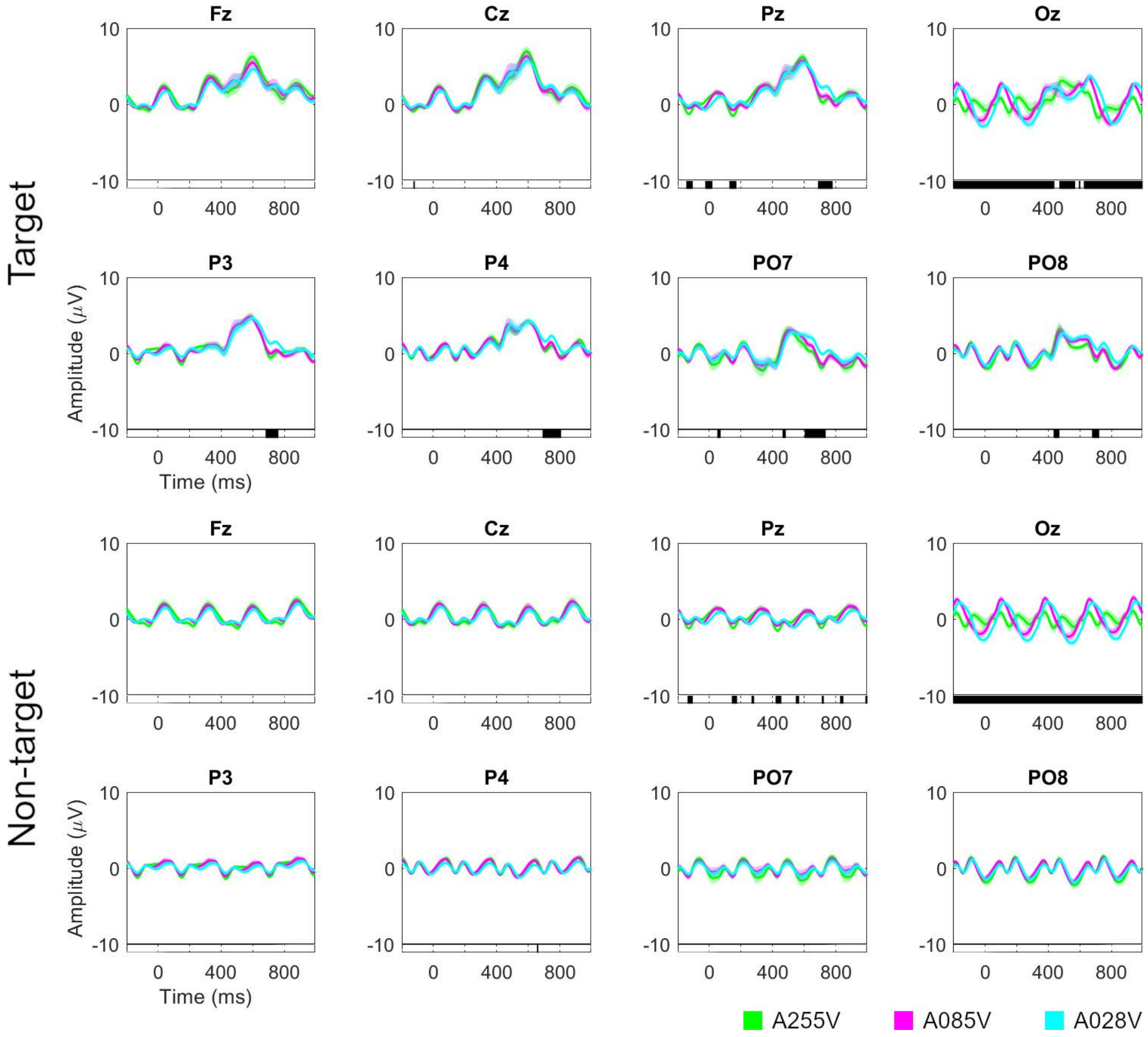

Figure 2. Grand average event-related potential waveforms for the target and non-target stimuli signals for all channels used (Fz, Cz, Pz, Oz, P3, P4, PO7, and PO8) and for the three conditions varying in the level of stimulus transparency in the brain-computer interface (BCI) task of Session 2 (A255V, A085V, and A028V). Significant intervals are denoted on the bottom line of each plot. The Benjamini-Hochberg procedure was applied to control the false discovery rate and minimize type I errors by adjusting the *p*-value.
